# Progression from Type 2 Macular Neovascularization to Fibrovascular Pigment Epithelial Detachment

**DOI:** 10.3390/vision5020016

**Published:** 2021-03-25

**Authors:** Hoang Mai LE, Gérard Mimoun, Salomon Y. Cohen, Camille Jung, Oudy Semoun, Eric H. Souied

**Affiliations:** 1 Department of Ophthalmology, Centre Hospitalier Intercommunal de Créteil and University Paris Est, 94000 Créteil, France; gemimoun@gmail.com (G.M.); sycsyc75@gmail.com (S.Y.C.); oudysemoun@gmail.com (O.S.); eric.souied@chicreteil.fr (E.H.S.); 2 Retina Center of Ecole Militaire, 75007 Paris, France; 3 Ophthalmic Center for Imaging and Laser, 75015 Paris, France; 4 Centre de Recherche Clinique-Centre de Ressources Biologiques, Intercity Hospital, 94000 Créteil, France; camille.jung@chicreteil.fr

**Keywords:** age-related macular degeneration, angioid streaks, fibrosis, fibrovascular pigment epithelial detachment, macular neovascularization, myopia

## Abstract

The aim of this study was to report unusual progression of type 2 macular neovascularization (MNV) associated with age-related macular degeneration (AMD), high myopia or angioid streaks. Retrospective multicentric observational case series data were used. Eyes that progressed from type 2 MNV secondary to AMD, high myopia or angioid streaks to fibrovascular pigment epithelial detachment (PED) were included. A total of 29 treatment-naive eyes from 29 patients with type 2 MNV secondary to AMD (*n* = 14), high myopia (*n* = 10) or angioid streaks (*n* = 5) that progressed to a fibrovascular PED on Spectral Domain-Optical Coherence Tomography were used. This progression occurred within 3 months after anti-VEGF therapy initiation. Logarithm of minimum angle of resolution (LogMAR) visual acuity improved significantly after anti-VEGF therapy, from 0.55 (SD ± 0.30) (20/63–20/80) at baseline to 0.30 (20/40) at 3 months, and 0.33 (20/40) at the final follow-up (mean follow up: 3.68 years). Mean number of intravitreal injections per year for patients with a total follow-up ≥ 12 months (*n* = 24) was 4.3 ± 2.1 per year. Progression from type 2 MNV to a fibrovascular PED may occur in patients suffering from AMD, high myopia or angioid streaks. This progression appears early after initiation of anti-VEGF therapy and is associated with a favorable visual and anatomical outcome, at least on a short follow up basis.

## 1. Introduction

In the Western world, age-related macular degeneration (AMD) is the leading cause of visual loss in patients older than 50 [1,2]. In neovascular AMD, ingrowth of macular neovascularization (MNV) is responsible for severe visual loss due to exudation, bleeding or scarring [3].

Over the last few decades, anti-VEGF therapy has revolutionized the treatment of several retinal diseases. To date, anti-VEGF intravitreal injection is the most commonly performed ophthalmic procedure for the treatment of different retinal diseases such as neovascular AMD, diabetic retinopathy, retinal vein occlusion and secondary neovascularization in tumoral or inflammatory diseases [4,5,6,7,8]. 

In neovascular AMD, it has been shown that functional prognosis worsens if treatment by anti-VEGF intravitreal injections is delayed and strongly correlates with visual acuity at the time of the first injection [9]. Various molecules are available (ranibizumab, aflibercept and bevacizumab) and all have been proven to be safe and efficient for controlling MNV [4]. 

The common classification of neovascular AMD based on fundus fluorescein angiography (FA) was established by the Macular Photocoagulation study which characterized lesion subtypes as “classic” or well-defined MNV, and “occult” or ill-defined MNV [9]. Later, with the advent of Spectral Domain-Optical Coherence Tomography (SD-OCT), visualized anatomic subtypes of neovascularization were defined as type 1 MNV (sub-epithelial), type 2 MNV (pre-epithelial) and type 3 MNV (intravascular proliferation) [10,11]. 

Among patients with neovascular AMD, Type 2 MNV is present in 9% to 17% of the cases [12]. Type 2 MNV is also a well-known complication of high myopia and, although the prevalence varies according to studies and ethnicities, it is estimated to be present in around 1.5% to 11.3% of this population [13,14,15,16] Angioid streaks can be also be complicated by type 2 MNV, occurring in approximately 72%–86% of the cases [17,18]. Type 2 MNV appears on SD-OCT as a hyperreflective band located in the subretinal space, above the retinal pigment epithelium (RPE). It is usually associated with disorganization of the inner segments/outer segments (IS/OS), and in some cases with intra retinal cysts [11,19]. In the large majority of cases, large serous or typical fibrovascular retinal pigment epithelial detachment (PED) is not observed, although a small sub-RPE component can be noticed [19]. In contrast, type 1 MNV is typically associated with the presence of an elevation or a detachment of the RPE and appears as a sub-RPE lesion [20,21]. Various changes can appear in the RPE band, such as a hyperreflectivity, a fragmentation or a thinning. The neovascular complex can appear as a moderately reflective material in the sub-RPE space [21]. Progression from type 1 MNV to fibrovascular PED is a well-known evolution in neovascular AMD patients. Recently, a case series study consisting of nine neovascular AMD patients reported a peculiar anatomic evolution of type 2 MNV after early treatment with intravitreal anti-vascular endothelial growth factor therapy (anti-VEGF) [22]. We also observed this peculiar pattern of progression in some patients suffering from type 2 MNV secondary to AMD, but also secondary to other macular disorders such as pathologic myopia and angioid streaks. These patients presented with a fibrovascular PED early after initiation of anti-VEGF therapy. 

The purpose of the study was to describe in SD-OCT this anatomic peculiar pattern of progression from type 2 MNV to fibrovascular PED in a large series of patients treated with anti-VEGF therapy in the context of type 2 MNV secondary to AMD, but also pathologic myopia, or angioid streaks.

## 2. Materials and Methods

### 2.1. Study Population

This multicentric observational retrospective case series study was conducted at the Centre Hospitalier Intercommunal de Créteil (Créteil, France) and at the Ecole Militaire Retina Center (Paris, France). The study design was approved by the Federation France Macula Ethics Committee (2016–22). The described research methods and analysis adhered to the tenets of the Declaration of Helsinki. Clinical records and multimodal imaging data were retrospectively reviewed for eyes diagnosed with type 2 MNV that progressed towards fibrovascular PED seen in the clinic from November 2015 to November 2016.

Inclusion criteria included patient age > 18, treatment-naïve type 2 MNV at baseline (defined on the basis of FA and SD-OCT) secondary to AMD, pathologic myopia or angioid streaks that progressed towards a fibrovascular PED after anti-VEGF therapy. Additional inclusion criteria were ≥3 months of longitudinal reverse follow-up. Clinical and imaging data were analyzed at 12 months after the diagnosis when available and also at the end of the follow up if follow up > 12 months.

Exclusion criteria included a history of photodynamic therapy and/or evidence of type 1 or type 3 or fibrovascular PED at first initial examination on our medical imaging records. 

All patients initially underwent a complete examination that included the best-corrected visual acuity (BCVA) measured on a Snellen score chart (converted to logarithm of minimum angle of resolution (logMAR) for the statistical analysis), fundus examination, infrared monochromatic fundus and autofluorescence photographs, FA, indocyanine green angiography (ICGA) and SD-OCT (Retina Angiograph II and Spectralis; Heidelberg, Germany). FA and ICGA were assessed according to a standard scheme (imaging of 1 min or less was defined as the early phase; imaging around 5 min for FA and 30 min for ICGA was defined as the late phase).

Patients were treated with anti-VEGF therapy with an initial loading phase (3 monthly intravitreal anti-VEGF injections, either with ranibizumab 0.5 mg or aflibercept 2 mg) followed by a pro re nata phase (OCT-guided regimen). We reported the initial aspect of the type 2 MNV based on SD-OCT and FA, performed at initial examination in our center. On SD-OCT scans, we evaluated the presence of subretinal fluid, subretinal hyperreflective exudation, intra retinal cysts, and measured the central macular thickness (CMT). On fundus infrared and autofluorescence photographs, we noted the presence or the absence of a hyperreflective or hyperautofluorescent ring, respectively. 

### 2.2. Statistical Analysis

All data were collected in an Excel file (Microsoft Excel 2016). Statistical analysis was performed using the IBM SPSS statistics software using the Wilcoxon signed-rank test for quantitative data (age, duration of the follow-up, BVCA, number of intravitreal injections, CMT) and the McNemar test for nominal data (presence or absence of subretinal fluid, subretinal hyperreflective exudation and intraretinal cysts). A *p* value < 0.05 was considered statistically significant.

## 3. Results

### 3.1. Study Population

A total of 29 treatment-naive eyes from 29 patients (16 females, 13 males) with type 2 MNV secondary to AMD, angioid streaks or high myopia that progressed to a fibrovascular PED on SD-OCT during follow-up were included. A total of 14/29 eyes (48.3%) had neovascular AMD with a mean age of 79.9 years (range: 72–90 years), 10/29 eyes (34.5%) had high myopia with a mean age of 68.5 years (range: 42–80 years) and 5/29 eyes (17.2%) had angioid streaks with a mean age of 63.6 years (range: 56–76). Mean duration of the follow-up was 3.68 years (SD ± 2.41, range 0.25–8); 24/29 patients (82.7%) had a follow-up ≥ 12 months and 20/29 patients (68.9%) had a follow-up ≥ 36 months.

### 3.2. Visual Acuity

Mean BCVA expressed in LogMAR was 0.55 ± 0.30 (20/63–20/80) at baseline. A total of 3 months after the first anti-VEGF injection, mean BCVA improved significantly to 0.30 ± 0.23 (20/40) (mean difference = 0.26 (2 lines and 3 letters of improvement), IC95% (0.17–0.34), *p* < 0.0001). Similarly, at 1 year, mean BVCA was 0.35 ± 0.30 (20/40), showing a significant improvement compared to baseline (mean difference 0.16 ± 0.25; IC95% (0.06–0.27), 1 line and 2 letters of improvement) (*p* = 0.004). Final mean BVCA for patients with a follow-up > 1 year after the diagnosis (*n* = 24) was 0.27 ± 0.27 (20/40) (with a mean total follow-up of 4.85 years). This was a significant improvement compared to baseline, with a mean difference of 0.25 (IC95% (0.12-0.38) *p* = 0.001, 2 lines and 2 letters of improvement). Mean number of intravitreal injections per year for patients with a total follow-up ≥ 12 months (*n* = 24) was 4.3 ± 2.1 per year. 

### 3.3. Anatomical Outcome

**Figure 1:**
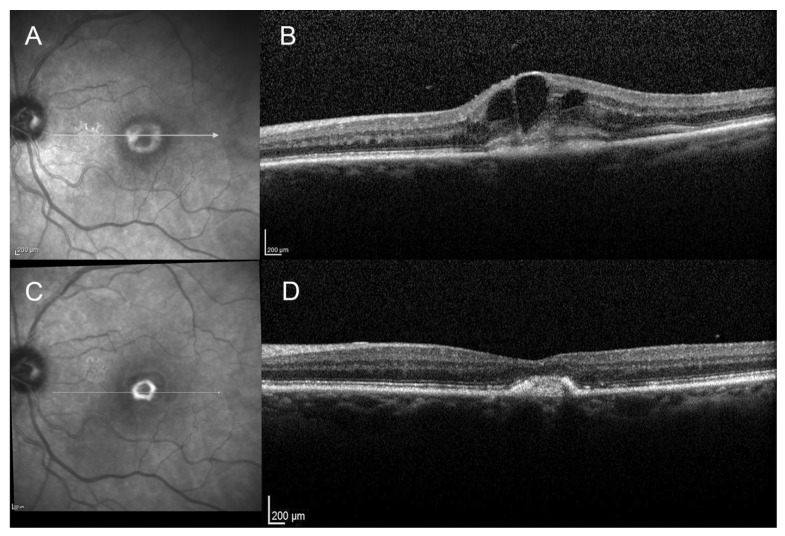
Spectral domain optical coherence tomography (SD-OCT) scans of a 79-year-old female patient with wet age-related macular degeneration (AMD), at baseline and at 3-month follow-up. (**A**): Infrared reflectance photography at baseline showing a hyperreflective foveal ring. (**B**): SD-OCT scan at baseline, showing a pre-epithelial neovascular lesion (type 2 lesion) with subretinal hyperreflective exudation and intra retinal cysts. (**C**): Infrared reflectance photography at 3-month follow-up, showing the strengthened and well demarcated hyperreflective ring. (**D**): SD-OCT scan at 3-month follow-up showing a hyperreflective subretinal lesion, forming the fibrovascular pigment epithelial detachment (PED).

**Figure 2:**
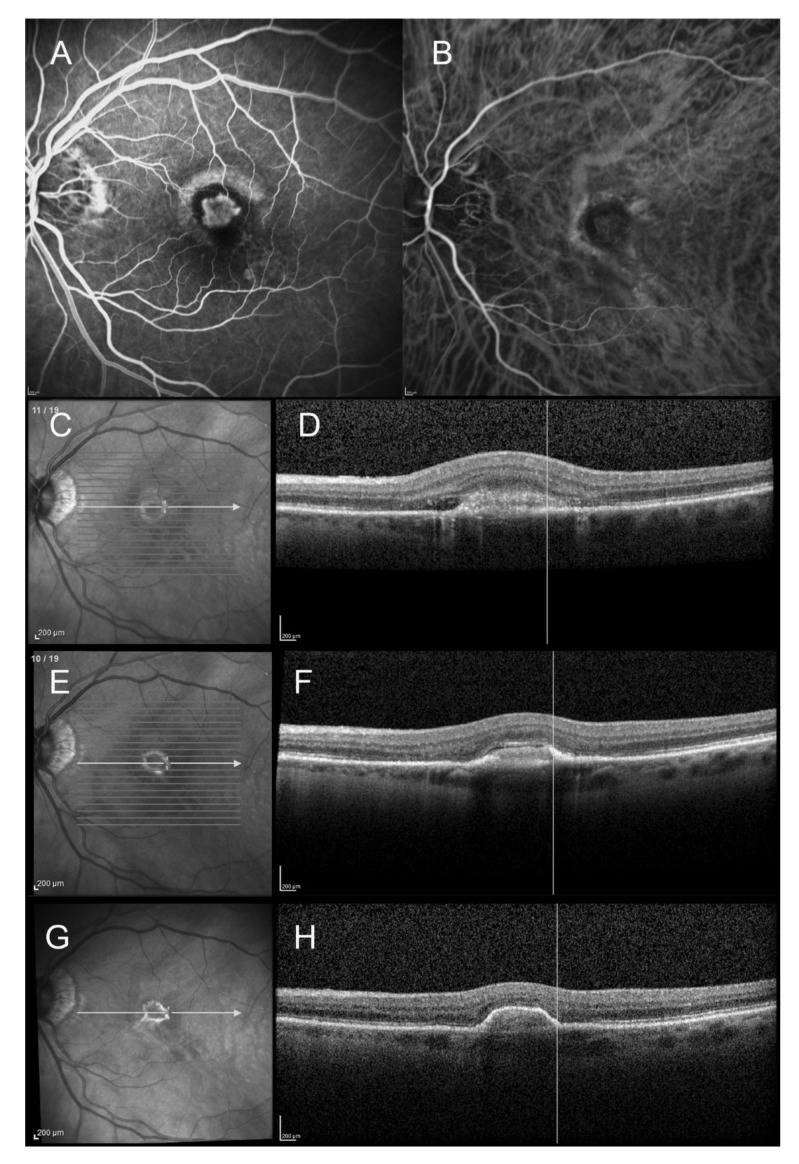
Spectral domain optical coherence tomography (SD-OCT) findings of an 83-year-old female patient with wet age-related macular degeneration (AMD) and initial type 2 macular neovascularization (MNV). The SD-OCT scans show the progression from type 2 MNV to fibrovascular PED and its margins (at baseline, 1 month after the first intravitreal anti-VEGF injection, and at 1-year follow-up). (**A**): Early-phase fluorescein angiogram (23 s) at baseline showing a well-demarcated early hyperfluorescence surrounded by a hypofluorescent rim. (**B**): Early-phase indocyanine green angiogram (21 s) at baseline showing the neovascular network surrounded by hypocyanescent margins. (**C**): Infrared reflectance photography at baseline, showing the initial hyperreflective ring. (**D**): Corresponding SD-OCT scan, showing the initial type 2 MNV, with subretinal fluid and hyperreflective exudation. The hyperreflective ring is within the limits of the neovascular lesion. (**E**): Infrared reflectance photography at 1 month after the first anti-VEGF injection, showing that the hyperreflective ring appeared well-demarcated and retractile. (**F**): Corresponding SD-OCT scan: the fibrovascular PED can be observed. (**G**): Infrared reflectance photography at 1-year follow-up: the hyperreflective ring is smaller and has a retractile aspect. (**H**): SD-OCT scan at 1-year follow-up: the hyperreflective ring corresponds to the limits of the fibrovascular PED lesion.

**Figure 3:**
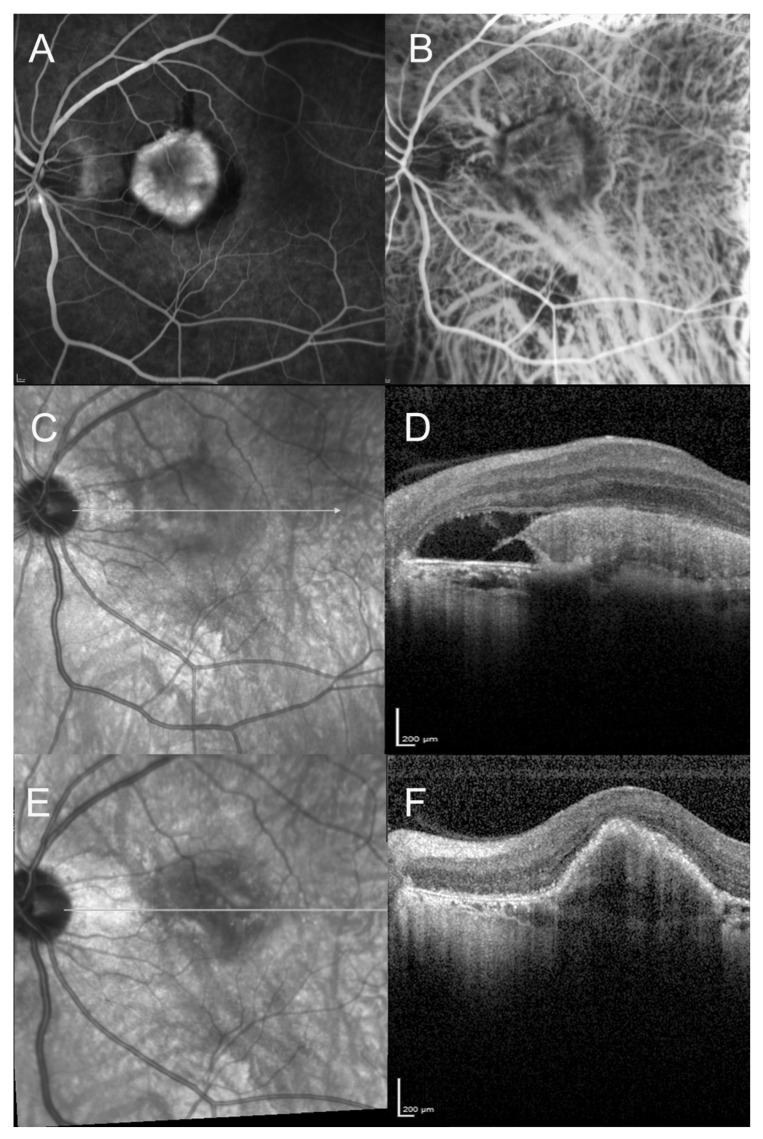
A 64-year-old man diagnosed with high myopia, presenting with visual acuity loss (20/100). (**A**–**D**): Fluorescein angiography, indocyanine green angiography, Infrared reflectance photography, and horizontal Spectral domain optical coherent tomography (SD-OCT) at baseline. Neovascular lesion in a myopic eye with tessellated fundus. SD-OCT scan showing the hyperreflective pre-epithelial neovascular lesion, with subretinal fluid. We can note the disruption of the retinal pigment epithelium (RPE) layer and the alteration of the photoreceptors layer. (**E**,**F**): Infrared reflectance photography and horizontal scan of the SD-OCT at 1-year follow-up, after anti-VEGF therapy. The fibrovascular PED can be observed. Visual acuity improved to 20/40.

**Figure 4:**
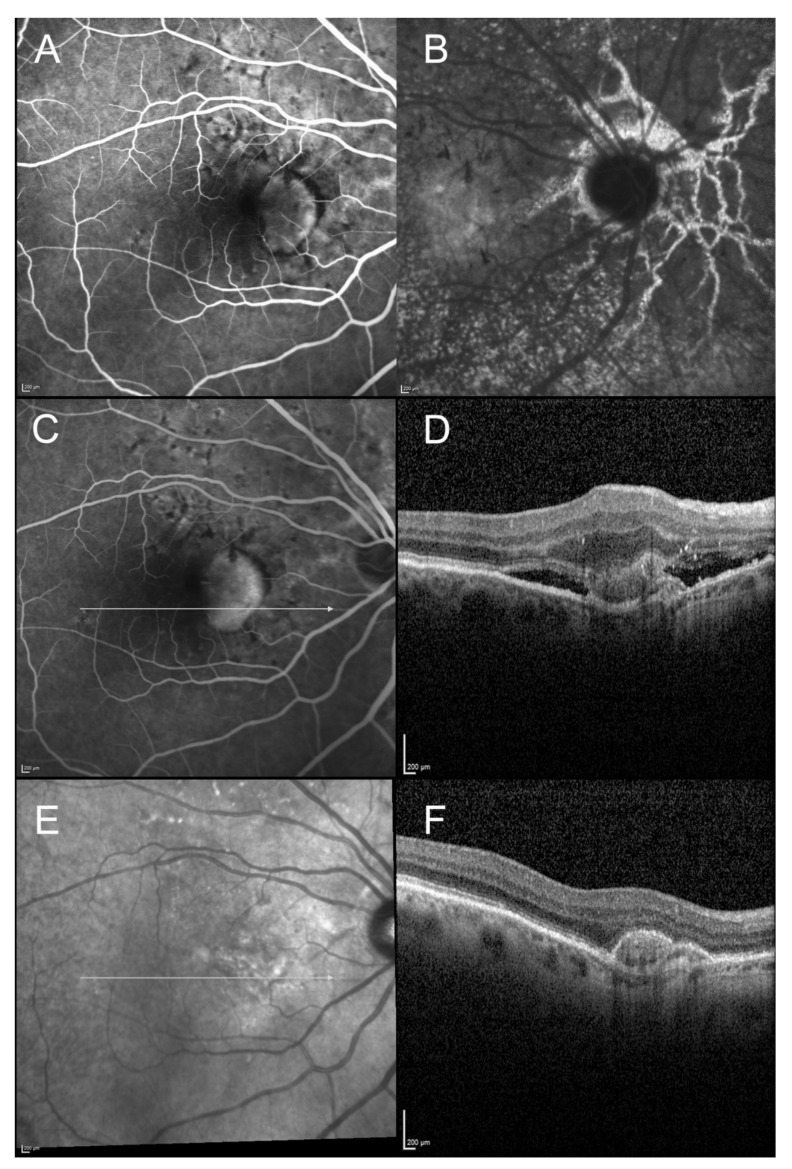
Imaging of a 64-year-old woman diagnosed with angioid streaks, presenting with visual acuity loss. (**A**): Fluorescein angiography (22 s) showing the well demarcated hyperfluorescent type 2 neovascular lesion. (**B**): Indocyanine green angiography (late phase): visualization of the type 2 neovascular lesion is less obvious, but the late phase shows the angioid streaks network clearly. (**C**,**D**): Fluorescein angiography and horizontal scan of the spectral domain optical coherence tomography (SD-OCT) at baseline. Well demarcated hyperfluorescent type 2 neovascular lesion, with subretinal fluid and hyperreflective exudation observed on SD-OCT. (**E**,**F**): Infrared reflectance photography and horizontal scan of the SD-OCT, at 1-year follow-up. SD-OCT scan shows the fibrovascular PED without any exudation signs.

On the initial SD-OCT, all patients (100%) presented with a highly reflective subretinal lesion above the RPE with a fusiform thickening and disruption of the RPE and choriocapillaris reflectivity, consistent with type 2 MNV (Figure 1, Figure 2, Figure 3 and Figure 4). The diagnosis of type 2 MNV was confirmed in all cases by FA and ICGA. Type 2 MNV was identified as a well-demarcated lacy area of hyperfluorescence during the early-phase angiogram accompanied by a progressive leakage in the overlying subsensory retinal space during the late phases of the angiogram. On ICGA, an area of hyperfluorescence corresponding to the choroidal neovessel structure was seen during the early phase. 

At baseline, 18 eyes had subretinal fluid (62.1%) and 22 eyes had a subretinal hyperreflective exudation (75.9%), but only 2 patients had an initial macular edema with intraretinal cysts (0.07%). PED was not observed in any of the included eyes at baseline (exclusion criteria). We noted a thickened retina in all cases with a mean CMT at 397 ± 102 μm. It is notable that none of these eyes presented a hemorrhage either at baseline or during follow-up. 

At baseline, infrared imaging showed a whitish ring surrounding a central dark core in 37.9% of cases (*n* = 11). This has previously been described in patients with type 2 MNV in AMD [23]. The same proportion of patients (37.9%, *n* = 11) presented a hypo-autofluorescent area in the center of the lesion surrounded by a hyper-autofluorescent ring. If present, the hyperreflective and hyperautofluorescent ring were located in 90% of the cases not at the margins but within the limits of the regressed neovessels, as previously described by Dolz-Marco et al [22]. After anti-VEGF therapy, a subjective downward trend of the size of the hyperreflective ring was observed by retinal specialists (OS, GM) (non-measured) (Figure 2).

At final examination, SD-OCT showed a fibrovascular PED (Figure 1, Figure 2, Figure 3 and Figure 4). This progression pattern from type 2 MNV to fibrovascular PED was observed in patients with AMD (Figure 1), high myopia (Figure 3) and angioid streaks (Figure 4). In all cases, the content of the lesion was heterogeneous but predominantly hyperreflective. A significant decrease in central macular thickness, with a mean CMT of 340 ± 59 μm (mean difference = 46 μm, IC95% (11-82), *p* = 0.01) was observed at 1-year follow-up (*n* = 24/29 patients). There was also a marked decrease in the number of eyes presenting with subretinal fluid (*n* = 9, *p* = 0.01), and subretinal hyperreflective exudation (*n* = 3, 12.5%, (*p* < 0.001) at 1 year of follow-up. There was no significant change regarding the intraretinal cysts (*p* = 0.317) (present in only two patients at baseline). Longitudinal analysis of this progression pattern showed that it developed early in the course of the disease, within three months after initiation of intravitreal anti-VEGF therapy. Moreover, this pattern seemed to be stable over time as all patients for which follow-up was available ≥1 year (*n* = 24/29) still had this pattern one year after the first intravitreal injection of anti-VEGF. 

## 4. Discussion

In the present study, we observed progression from type 2 MNV to fibrovascular PED early after intravitreal injections of anti-VEGF therapy in patients with AMD, angioid streaks and high myopia. This pattern corresponds to a hyperreflective scar lesion, surrounded by a pigmentary reaction. On SD-OCT, we observed a hyperreflective layer that resembles the RPE on OCT, although no conclusions about the precise cellular structure of this layer can be drawn. Stern et al. first reported the development of a “pigmented hypertrophy” around the MNV lesion, accompanied with MNV regression, in three AMD patients treated with anti-VEGF therapy [24,25]. These lesions were associated with a slower disease progression. Dolz-Marco et al [22]. recently described this pattern in a series of nine patients suffering from neovascular AMD. They defined the lesion as a regression of a type 2 lesion into a type 1 pattern associated with a better visual prognosis. In our series of 29 patients, we analyzed the data of treatment-naïve patients with pure type 2 neovascularization secondary to neovascular AMD, but also to angioid streaks and high myopia. We noticed that this progression pattern appeared early after initiation of anti-VEGF therapy, within the first 3 months of treatment. Overall, this pattern was associated with a good visual prognosis since mean visual acuity 3 months after the first injection and at 1 year remained >0.35 in logMAR (20/40–20/50). This good visual acuity was observed in most of the cases, 55% of the patients having a final visual acuity ≥ 20/32. In our series, the patients required a limited annual number of intravitreal injections of anti-VEGF agents with a mean of 4.3 ± 2.1 injections per year. From these results, we hypothesize that progression from type 2 MNV to fibrovascular PED is associated with a good visual prognosis, at least on short follow up basis. Results from a recent study confirmed that patients with MNV secondary to angioid streaks showed a regression or a stabilization of the lesions with a limited number of intravitreal anti-VEGF injections [26].

Whether this lesion is a final stage of the evolution of type 2 neovascularization after intravitreal anti-VEGF therapy is not known yet. However, it seems that the lesion is quite stable and may be a kind of scarring leading to a peculiar form of sub RPE fibrosis without atrophy. Indeed, in patients with a longer follow-up (>3 years), we noticed that the lesion had a white appearance on fundus examination. In those cases, the lesion on SD-OCT was hyperreflective and with well-determined boundaries. This peculiar form a fibrosis is different from fibroglial scars. 

With the advent of anti-VEGF therapy, a substantial regression of MNV has been made possible, with preservation of central vision in numerous cases. 

Nevertheless, some studies have shown a subsequent risk of evolution of the treated MNV towards macular atrophy or subretinal fibrosis [27,28]. This sub RPE fibrosis may be the consequence of either the natural healing process or of anti-angiogenic therapies. This progression pattern from type 2 MNV to fibrovascular PED observed in patients after anti-VEGF therapy could potentially correspond to an evolution towards a peculiar form of fibrosis, but with an unexpected good visual prognosis. Daniel et al. [29,30] observed in their prospective cohort study of 1059 eyes with AMD treated with anti-VEGF therapy that one third of the eyes developed a fibrous scar after 1 year of treatment, and one half after 2 years. Classic MNV was a risk factor for developing a scar and the risk tripled for predominantly classic MNV compared to occult MNV. Among eyes that showed a non fibrotic scar at 1 year, fibrotic scar developed in 5% at 2 years and 28% at 5 years. 

Although the human RPE forms early in eye development and rarely remodels throughout life, some studies have shown that RPE cells can be activated in vitro in the presence of a variety of growth factors capable of promoting their proliferation [19]. In vivo, using various animal models, RPE has been shown to have a good regenerative and wound healing capacity [25,28]. In contrast, human RPE proliferation occurs rarely and only in particular conditions, for example in proliferative vitreoretinopathy secondary to retinal detachment [31,32,33]. In these cases, RPE proliferation is often destructive, accompanied with epithelial–mesenchymal transition, and is associated with a worse visual prognosis. In this series of patients, this progression pattern seemed to be associated with a controlled proliferative response, which could perhaps have a repairing role.

The exact nature of the pigmentary reaction, its precise structure and function, are still uncertain. Although this pattern of progression is delimited by a layer that shares the same characteristics on SD-OCT with the RPE, it seems unclear how the original RPE monolayer with its adherent underlying Bruch’s membrane could migrate on top of the MNV. Perhaps, a tissue layer that resembles the RPE on SD-OCT develops on the surface of the MNV and gives the illusion that the MNV has become sub-RPE. 

This study has several limitations, among them its retrospective nature. Due to the design, we cannot estimate the frequency of this peculiar course. Moreover, due to the lack of histological analysis, we can only speculate on the mechanism of this peculiar scarring process. It could correspond to a pigmented reaction. Indeed, rapidly after the start of the anti-VEGF therapy, the inactive choroidal neovessels seem to be enveloped by a surrounding pigmentary reaction, forming a dome shape on SD-OCT. This pigmentary reaction, of which precise histopathologic the characteristics are still unknown, seems to be associated with a good visual prognosis, with lower chances of exudative recurrences.

Whatever its origin, the pattern should be recognized because it corresponds to a healing process with, usually, retention of a good visual prognosis. Further studies would be needed to confirm our results: a control group with patients presenting with type 2 neovascularization that did not progress towards a fibrovascular PED and a longer follow-up would be needed to confirm the stability of such lesions. A larger sample size would also help to increase the evidence about the reported data.

In conclusion, progression from type 2 MNV to fibrovascular PED can occur in different macular disorders, such as AMD, high myopia or angioid streaks. It is associated with a favorable visual and anatomical outcome. An early diagnosis could be of interest, as a prognosis factor.
